# Arctic–Eurasian climate linkage induced by tropical ocean variability

**DOI:** 10.1038/s41467-019-11359-7

**Published:** 2019-08-01

**Authors:** Shinji Matsumura, Yu Kosaka

**Affiliations:** 1 Faculty of Environmental Earth Science, Hokkaido University, Kita 10 Nishi 5, Kita-ku, Sapporo 060-0810 Japan; 2 Research Center for Advanced Science and Technology, University of Tokyo, 4-6-1 Komaba, Meguro-ku, Tokyo 153-8904 Japan

**Keywords:** Climate sciences, Atmospheric science, Climate change, Cryospheric science, Ocean sciences

## Abstract

Eurasian continent has experienced cold winters over the past two decades in contrast with Arctic warming. Previous studies have suggested that the cold Eurasian winters are associated with Arctic sea-ice loss, while others attributed them to atmospheric internal variability. However, here we show that the Arctic and Eurasian climate linkage is driven by the combination between atmospheric teleconnection originating in the tropical oceans and Arctic sea ice. Like a battery charges a capacitor, El Niño heats the tropical Atlantic, and the warmer Atlantic condition persists until early winter of El Niño-decay year. We find that the persisting tropical Atlantic warming induces anomalous Rossby wave train arching to Eurasia, leading to Arctic sea-ice increase and Eurasian warming. In La Niña phase these changes are reversed. Our results therefore suggest that the combination of recent tropical Pacific cooling and Arctic sea-ice loss have contributed to the frequent Eurasian cold winters.

## Abstract (web summary)

El Niño warms the tropical Atlantic, which in turn induces an anomalous Rossby wave train, triggering Arctic sea-ice growth and Eurasian warming in the El Niño decay year. This teleconnection via the tropical Atlantic and the Arctic in La Niña decay year contributes to Eurasian cold winter extremes.

## Introduction

Despite ongoing global warming, number of continuously freezing days has increased and minimum temperatures have decreased over northern mid-latitude continents since the 1990s, while the frequency of unusually cold winter months in Eurasia markedly decreased from the 1980s to 1990s^1^. This trend reversal in cold extremes is associated with the development of Siberian high^2–4^ that leads to cold advection and frequent occurrence of cold events over Eurasia. Arctic sea-ice reduction is suggested as the driver of the recent trend, but mechanisms underlying this Arctic–Eurasian climate linkages proposed by many previous studies is yet inconclusive^1,5–10^. The impact of Arctic sea ice variations on mid-latitude climate is a topic of much debate.

Meanwhile, the Arctic climate variation is not necessarily closed within the Arctic and high-latitude regions. Several studies have implicated fluctuating sea surface temperatures (SST) outside the polar cap as an important driver of the recent Arctic warming^8,11^. In particular, tropical Pacific SST variability, which is regarded as a key pacemaker of surface global warming^12,13^, is suggested as a cause of recent surface warming in Greenland through atmospheric teleconnection^14^. Here we show that a combination of atmospheric teleconnection originating in the tropical oceans and Arctic sea-ice variability is a driver of the Arctic–Eurasian climate linkage. This study focuses on early winter (OND: October–December) during the freezing season, when recent Arctic warming is most pronounced and the impact of Arctic sea-ice loss on the atmosphere is likely to maximise^11^. Seasons refer to those in the Northern Hemisphere.

## Results

### Observed variations after ENSO decay

El Niño usually develops during summer and autumn, peaks during early winter, and decays through the following spring. El Niño heats also the tropical Atlantic and Indian Oceans by the tropical tropospheric warming and atmospheric bridges, like a battery charges a capacitor^15–17^. The persisting tropical Indian Ocean warming affects western Pacific climate like a discharging capacitor during summer when the equatorial Pacific signal of El Niño has dissipated^18^. Furthermore, this Indian Ocean capacitor effect extends to the following September–November (SON) by maintaining equatorial wave response to isolated heating on the equator^19,20^ (Supplementary Fig. 1). Indeed, these atmospheric responses in SON in the decay year of El Niño–Southern Oscillation (ENSO) are distinct from those in the ENSO-developing year (Supplementary Fig. 2). Here we denote the ENSO index at preceding and subsequent boreal winters as ENSO(–1) and ENSO(0), respectively (the lagged autocorrelation of the ENSO index is close to unity from summer though subsequent winter^18^).

**Fig. 1:**
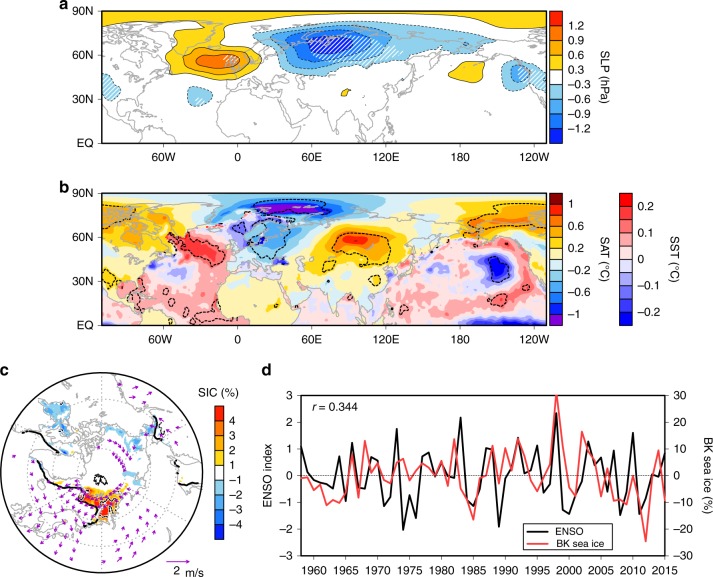
Observed climate anomalies in early winter of El Niño-decay year. Regressed anomalies of **a** sea level pressure (SLP), **b** surface air temperature (SAT) and sea surface temperature (SST) and **c** sea ice concentration (SIC) (shading) and 925-hPa wind velocity (vectors; limited to 50°–82°N) in early winter onto the El Niño–Southern Oscillation (ENSO) index in preceding winter (ENSO(–1)). SAT is shown over land or ice while SST is shown over open ocean in **b**. Hatching in **a** and black-dotted contours in **b**, **c** indicate statistical significance (for SIC in **c**) at the *p* < 0.05 level, and thick black contours in **c** indicate mean SIC of 10%. **d** Time series of the detrended ENSO index (1-year forward shift) and SIC anomalies averaged in the Barents–Kara (BK) Seas (70°–80°N, 50°–90°E)

Interestingly, another atmospheric wave train is found over the North Atlantic and northern Eurasia in SON of ENSO-decay year, which persists into early winter (Fig. 1a). Of noteworthy is the lower sea level pressure (SLP) over northern Eurasia, indicating the weakened Siberian high. This leads to Eurasian warming in its southeastern flank through anomalous warm advection and Arctic cooling by anomalous cold advection over the Barents–Kara (BK) Seas^2^ (Fig. 1b). These features are confirmed in other reanalysis and reconstruction data sets (Supplementary Fig. 3). The BK sea-ice concentration (SIC) indeed increases during the freezing season following El Niño decay (Fig. 1c), supporting that BK SIC variability is associated with atmospheric variability^21^. The correlation between BK SIC and ENSO(–1) is 0.344 (Fig. 1d), significant at the 99% confidence level, while ENSO(0) correlation is close to zero. Although moderate, this significant BK SIC correlation with ENSO(–1) exceeds the persistence of summer BK SIC anomalies in early winter (Supplementary Fig. 4), as the persistence of Arctic sea-ice anomalies dramatically decreases from summer to autumn in only a few months^22^. In the North Atlantic, SST warms near 60°N, in the tropics, and along the eastern coast of the North Atlantic (Fig. 1b), resembling the major mode of the North Atlantic SST variations in autumn and winter^23,24^. In ENSO(0), however, these features including the Arctic SIC increase and the North Atlantic SST pattern are missing (Supplementary Fig. 2). Another strong SST cooling is also prominent in the northeast Pacific, with a cyclonic anomaly to the east (Fig. 1a), reminiscent of the recent warm blob when the sign is flipped^25,26^.

### Two major teleconnections

**Fig. 2:**
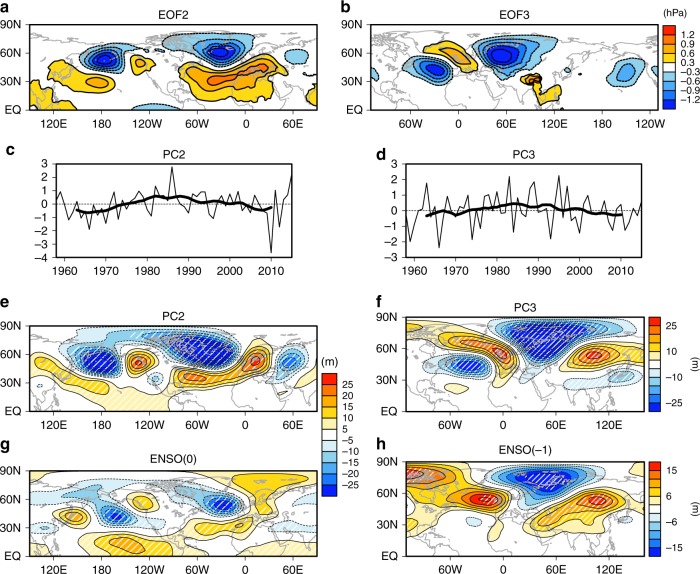
Two major modes of Northern Hemisphere circulation in early winter. **a** EOF-2 and **b** EOF-3 of early winter sea level pressure (SLP) and **c**, **d** corresponding principal components (PCs) for 1958–2015, respectively. Thick black lines in **c**, **d** indicate 11-year running means. Regressed anomalies of 300-hPa geopotential height onto PCs of **e** EOF-2 and **f** EOF-3, and **g** ENSO(0), and **h** ENSO(–1). Hatching in **e**–**h** indicates statistical significance at the *p* < 0.05 level

The distinct atmospheric and oceanic variations after ENSO dissipation are also supported by an empirical orthogonal function (EOF) analysis (see Methods). Figure 2 shows the second and third EOF modes (EOF-2 and EOF-3, respectively) of SLP over the Northern Hemisphere in early winter for 1958–2015 (explaining 15.2% and 11.5% of the total variance, respectively). The leading EOF mode represents the Arctic Oscillation, which explains 19.4% (Supplementary Fig. 5). The principal component (PC) corresponding to EOF-2 has an upward trend before the 1980s but a downward trend afterwards (Fig. 2c), intensifying in a negative phase. The EOF-3 resembles the wave-train pattern seen in Fig. 1a. Although this wave-train pattern has similar long-term changes to the EOF-2 (Fig. 2d), surface air temperature (SAT), SST and SIC anomalies are quite distinct (Supplementary Fig. 6). The EOF-2 pattern is related to a Pacific–North America (PNA)-like teleconnection combined with the North Atlantic Oscillation (NAO)^14^, while the EOF-3 pattern captures Arctic cooling, Eurasian warming and the North Atlantic SST variation as in Fig. 1b. The PC corresponding to EOF-3 is correlated with ENSO(–1), while EOF-2 is correlated with ENSO(0) (Supplementary Table 1). Although significant, the EOF-3 correlation with ENSO(–1) is moderate, consistent with a view that this anomaly pattern is an atmospheric internal mode whose occurrence is modulated by SST and sea ice anomalies^6,27^. Considering that the Arctic Oscillation (EOF-1) is mostly an internal atmospheric variability unforced by SST or sea ice^6^, these results suggest that ENSO(−1) can be a seasonal predictor of the BK sea ice and Eurasian SAT.

SAT variations are tied to upper-level atmospheric circulation. The PNA combined with NAO in the EOF-2 pattern can roughly explain ENSO(0) anomalies (Fig. 2e, g). The SLP EOF-3 is associated with a Rossby wave train that extends from the tropical North Atlantic towards the Arctic and eastern Eurasia (Fig. 2f), resembling the Eurasian teleconnection pattern^28^. ENSO(–1) also features a similar wave train from the North Atlantic across Eurasia (Fig. 2h). The combination of a trough over the BK Seas and a downstream anticyclone leads to Arctic cooling and Eurasian warming. These results demonstrate that the atmospheric circulation response to ENSO(–1) is completely different from that in ENSO(0), and suggest that the Arctic and Eurasian contrasting SAT anomalies are associated with ENSO(–1) and BK sea ice anomalies through the atmospheric circulation anomalies.

### The tropical Atlantic capacitor effect

**Fig. 3:**
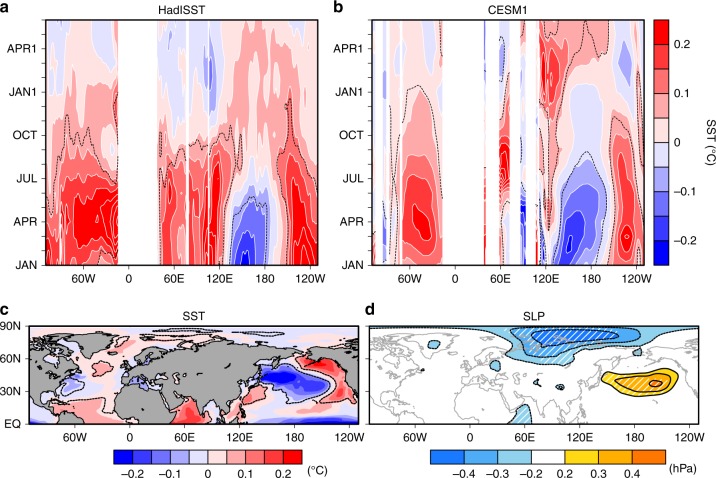
Simulated surface climate anomalies in El Niño-decay year. Longitude–time section of sea surface temperature (SST) anomalies over 10°–20°N regressed onto ENSO(–1) in **a** HadISST (1958–2015) and **b** CESM1 ensemble mean (1958–2013). The time evolution is from January of ENSO-decaying year through June of the subsequent year. Regressed anomalies of **c** SST and **d** sea level pressure (SLP) in early winter onto ENSO(–1) for CESM1 ensemble mean. Black dotted contours and hatching in **d** indicate statistical significance at the *p* < 0.05 level

What induces the early winter atmospheric circulation anomalies long after dissipation of the tropical Pacific signal of ENSO? ENSO charges the tropical Indian Ocean but also the tropical Atlantic^15,16,18^, as observed in early winter of ENSO-decay year (Fig. 1b). Although the warmer Indian Ocean condition persists from the El Niño peak through September, the tropical Atlantic warming further lingers until early winter (Fig. 3a). To confirm robustness of this persistent warming, we analyse a 10-member simulation of the Pacific Ocean-Global Atmosphere (POGA) pacemaker experiment^29^ where the tropical Pacific SST anomalies are restored towards observed counterparts. POGA reproduces the tropical Atlantic warming in a comparable magnitude with observations until early winter of ENSO-decay year (Fig. 3b, c). Furthermore, POGA successfully captures the Siberian high weakening (Fig. 3d), although Arctic sea-ice increase is not well simulated. A POGA simulation with another model shows that the tropical Atlantic warming is less persistent and the Siberian SLP anomalies are weak^13^, further supporting the key role of the tropical Atlantic warming to the Eurasian anomalies.

### Modelling evidence

**Fig. 4:**
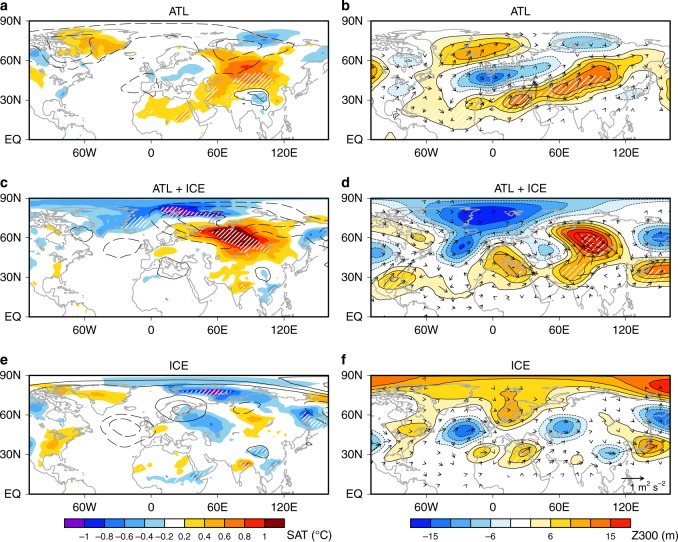
Simulated atmospheric responses to tropical Atlantic warming and Arctic sea ice increase. Surface air temperature (SAT) (shading) and sea level pressure (SLP) (contours for ±0.5, ±1, … hPa; solid for positive and dashed for negative) in **a** ATL (tropical North Atlantic warming)—CTL (control experiment) difference, **c** ATL + ICE (tropical North Atlantic warming and Arctic sea ice increase)—CTL difference, and **e** ICE (Arctic sea ice increase)—CTL difference. **b**, **d** and **f** As in **a**, **c** and **e**, respectively, but for 300-hPa geopotential height (Z300) (shading) and wave activity fluxes (vectors). Hatching indicates statistical significance at the *p* < 0.05 level for shaded anomalies

Although POGA successfully reproduces the lingering tropical Atlantic warming and its influence on the extra-tropics, a possibility remains that the extratropical North Pacific SST anomalies, which are stronger than in observations (Fig. 3c), are the driver of the Eurasian atmospheric circulation. To elucidate the tropical Atlantic role, we simulate atmospheric response to the tropical Atlantic warming in ENSO-decay year using an atmospheric general circulation model (Methods). A control (CTL) experiment consists of a 50-year integration forced by climatological SST and SIC. We have performed a tropical Atlantic (ATL) experiment forced by SST anomalies in the tropical North Atlantic (0°–30°N) in ENSO-decay year superposed on the global climatology. ATL—CTL difference (Fig. 4a) reproduces the observed Eurasian warming with weakened Siberian high in response to the tropical North Atlantic warming (Fig. 1a, b). In the upper troposphere, it captures anticyclonic anomalies over Eurasia, but the anomalous Arctic trough is missing (Fig. 4b).

We further examine impact of the BK sea ice increase on atmospheric circulation by additional two experiments (ICE and ATL + ICE). They are forced by BK SIC anomalies in ENSO-decay year (Fig. 1c) solely (ICE) and in combination with the tropical North Atlantic SST anomalies as in ATL (ATL + ICE) (see Methods). (ATL + ICE)—CTL difference presents amplified Eurasian warming with Siberian high weakening, producing the observed dipole of Arctic cooling and Eurasian warming (Fig. 4c) in a comparable magnitude with observations (Fig. 1b). The Eurasian warming in ATL + ICE is significantly stronger than in ATL, highlighting an amplifying role by the BK sea ice anomalies. Compared with ATL, the geopotential height response better captures the observed wave train from the tropical Atlantic towards eastern Eurasia via the Arctic (Fig. 4d). By contrast, ICE—CTL difference shows cooling only over the ice-increased BK Seas and cannot produce the Arctic-Eurasian linkage (Fig. 4e, f). Besides, the Arctic cooling in ICE is considerably weaker than in ATL + ICE. This indicates an important contribution of the Siberian high weakening to the Arctic cooling, in support of atmospheric driving of Arctic sea-ice variability^2,21,30^.

Modelling studies generally underestimate recent Eurasian cooling^8–10,27^, often despite excessively strong sea ice anomalies prescribed^6,31^, suggesting dominant role of internal atmospheric variability for Eurasian cooling. Our experiments also demonstrate that BK sea ice anomalies solely cannot induce strong Eurasian climate anomalies (Fig. 4e). However, ATL + ICE reproduces the Arctic and Eurasian SAT linkage (Fig. 4c). Despite that we do not artificially inflate prescribed SST or SIC anomalies, the Arctic–Eurasian SAT linkage is comparable in magnitude to observations. We therefore suggest that the Arctic–Eurasian climate linkage is driven by the combination between atmospheric teleconnection originating in the tropical oceans and Arctic sea ice.

**Fig. 5:**
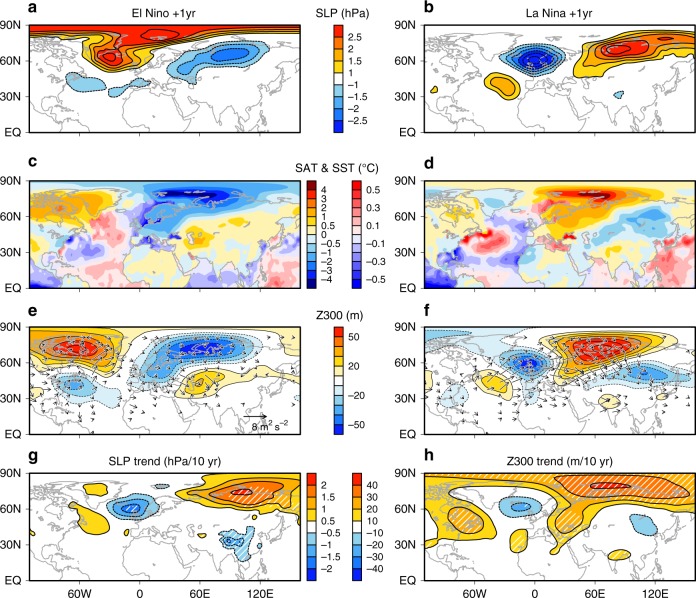
Composite mean differences in El Niño and La Niña decay-years and recent trends. Composite mean differences between El Niño-decay years (1992, 1995, 1998, 2003 and 2010) and climatology (1990–2015) in **a** sea level pressure (SLP), **c** surface air temperature (SAT), and sea surface temperature (SST), and **e** 300-hPa geopotential height (Z300) (shading). **b**, **d** and **f** As in **a**, **c** and **e**, respectively, but for La Niña-decay years (1999, 2000, 2008, 2011 and 2012). Vectors in **e**, **f** show the associated wave activity flux. Linear trend of **g** SLP (hPa decade^−1^) and **h** Z300 (m decade^–1^) from 1987 to 2015 (Hatching: *p* < 0.05 level)

Despite gradual weakening of the warmer tropical Atlantic condition throughout ENSO-decay year (Fig. 3a), SLP and precipitation anomalies undergo secondary peak in October over the warm pool around the Caribbean Sea (Supplementary Fig. 7), where climatological SST and precipitation reach a maximum in autumn^32^. The increased precipitation over the warm pool is reproduced by ATL + ICE (and also ATL), resulting from the tropical Atlantic SST warming. To the northwest of the increased precipitation, upper-tropospheric anticyclonic anomalies intensify in both observations and model (Supplementary Fig. 7), consistent with the characteristics of a Rossby wave response to enhanced latent heating. The intensified anticyclonic anomalies act as a Rossby wave forcing, propagating the wave activity northeastward, which lead to the Eurasian wave train in early winter (Fig. 4d). Although the observed wave train appears to propagate from the extratropical North Atlantic (Fig. 2h), the enhanced baroclinic atmospheric response over the tropical Atlantic is evident in SON (e.g., Supplementary Fig. 1). By a composite analysis, the Rossby wave train from the tropical Atlantic towards Eurasia via the Arctic is more pronounced (see Fig. 5). As only tropical Atlantic SST or Arctic sea ice cannot produce the Arctic–Eurasian SAT linkage (Fig. 4a, e), these results suggest that tropical Atlantic SST induces a Rossby wave and Arctic sea ice maintains or amplifies the wave activity. The extratropical North Atlantic SST anomalies may also play a role in amplifying the wave train^33^, where the positive SLP anomaly is likely to enhance northern European cooling through anomalous northerlies (Fig. 1a, b).

To support our observational findings and modelling results, we turn to Atmospheric Model Intercomparison Project (AMIP)-type experiment data (see Methods). Two model ensembles during the past 50 years simulate Arctic cooling and Eurasian warming with weakened SLP reasonably well in early winter of ENSO-decay year (Supplementary Fig. 8), although the response is weaker. We conclude that the coupling between tropical oceans and Arctic sea ice through atmospheric circulation plays a substantial role in Arctic and Eurasian climate linkage.

## Discussion

The impact of Arctic sea ice on Eurasian winter climate is highly nonlinear^34^ and considerably different among models^5–10^. Our results here demonstrate that without tropical forcing, the linkage between Arctic sea ice change and Eurasian climate would have been much weaker. The combination of tropical Atlantic SST and Arctic sea ice anomalies induces a stronger remote influence and improves the signal to noise ratio. Our result thus poses a novel perspective on the potential remote impact of Arctic sea ice variability and changes.

A composite analysis in recent El Niño- and La Niña-decay years shows that the Arctic and Eurasian climate anomalies are roughly in the opposite polarities at surface (Fig. 5a–d). This is consistent with our additional experiment driven by the difference between the negative (i.e., La Niña, tropical Atlantic cooling and BK sea ice decrease) and positive phases (Supplementary Fig. 9). However, the North Atlantic wave path is different between the two decay years (Fig. 5e, f), and the Arctic and Eurasian climate linkage appears to be more pronounced in decay years of La Niña than El Niño, possibly due to the asymmetry in their durations^35^. Recent atmospheric circulation trends (Figs. 2d and 5g, h) are consistent with the phase of La Niña-decay years. This result suggests that the combination of the recent tropical Pacific cooling^12,14^ and Arctic sea-ice loss have contributed to the frequent Eurasian cold winters, implying an interaction between natural climate variability and human-induced climate change^36^. The combination between tropical ocean variability and Arctic sea-ice change may help improvements of Arctic and Eurasian climate projections.

## Methods

### Observational data and analyses

Atmospheric data are from the Japanese 55-year reanalysis (JRA55)^37^, the ERA-Interim reanalysis^38^, and the NCEP Climate Forecast System Reanalysis (CFSR)^39^. SST and sea ice data are obtained from the Hadley Centre Global Sea Ice and Sea Surface Temperature (HadISST)^40^. Precipitation data are from GPCP^41^ and terrestrial surface air temperature data are from University of Delaware^42^. Sea level pressure data are also obtained from Hadley Centre Sea Level Pressure dataset (HadSLP2)^43^. This study focuses on the period 1958–2015, for which JRA55 is available. We confirm that the results here are independent of choice of reanalysis data and the results based on Arctic sea-ice data are consistent with those during the satellite era 1979–2015 (Supplementary Fig. 4). We refer to winter (November–January) SST averaged over the eastern equatorial Pacific (Nino-3.4: 5°S–5°N, 120°–170°W) as the ENSO index^18^. To determine the major modes of SLP variations, we performed an empirical orthogonal function (EOF) analysis of early winter (OND) SLP poleward of the equator. Before the EOF analysis, SLP anomaly data were weighted by the cosine of latitude to ensure that equal areas were afforded equal weight in the analysis. A wave activity flux that represents stationary Rossby wave propagation is based on ref. ^44^. All regression and correlation analyses are conducted after linear detrending, and the ENSO index is normalised. The significance test used in this study is a standard two-tailed *t*-test with degrees of freedom based on number of years.

### Sensitivity experiments with an atmospheric model

We have used the US Geophysical Fluid Dynamics Laboratory (GFDL) Atmospheric Model version 2.1 (AM2.1) with 24 vertical layers and ~200 km horizontal resolution. Atmospheric model simulation has the distinct advantage that SST and sea ice can be perturbed in a controlled way, to isolate its influence on the atmosphere^36^. In CTL experiment, the model is forced by climatological SST and sea ice for the period 1958–2015 and integrated for 50 years after 1-year spinup. ATL, ICE and ATL + ICE experiments branch off from CTL at January 1st of each year, thereby comprising 50-member ensembles. SST anomalies in the tropical North Atlantic (0º–30ºN, from the west to east coasts, with 10º linear tapering zones north and south) from January to December and SIC anomalies in the Norwegian and BK Seas (10º–110ºE, from the Eurasian coast to 90ºN, with 10º linear tapering zones east and west) from September to December of ENSO-decay year, obtained as regression anomalies onto ENSO(–1), are superposed to the global climatology for ATL and ICE, respectively, while both anomalies are superposed for ATL + ICE. Each experiment is integrated for 1 year. Radiative forcing is fixed at the 1990 level.

### Tropical Pacific pacemaker experiments

We have used a 10-member POGA pacemaker simulation performed with the US National Center for Atmospheric Research (NCAR) Community Earth System Model version 1 (CESM1) for Fig. 3b–d. Details are found in ref. ^29^. We have additionally examined another 10-member tropical Pacific pacemaker simulation by GFDL Coupled Model version 2.1 (CM2.1)^13^. For each of the experiments, ensemble-mean anomalies are regressed onto the ENSO index. The data have been linearly detrended beforehand for consistency with other analyses.

### A multimodel dataset

We used AMIP experiment data provided by the US National Oceanic and Atmospheric Administration (NOAA)-Earth System Research Laboratory (ESRL) Physical Sciences Division. The experiments chosen apply the observed radiative forcing and specify the observed SSTs under an atmospheric model. Two model ensembles are considered here; a 17-member ensemble with the GFDL AM3 for 1958–2014 and a 16-member ensemble with the ESRL-Community Atmospheric Model version 5 (CAM5) for 1958–2015. We have used the ensemble mean anomalies for each model and regressed them onto the ENSO index. We have linearly detrended the data beforehand for consistency with other analyses.

## Supplementary information

Supplementary Information

## Data Availability

All observational data used in this study are publicly available and can be downloaded from the corresponding websites (the JRA55 reanalysis: https://jra.kishou.go.jp/JRA-55/index_en.html; the ERA-Interim reanalysis: https://www.ecmwf.int/en/forecasts/datasets/reanalysis-datasets/era-interim; the CFSR reanalysis: https://rda.ucar.edu/datasets/ds093.1/; University of Delaware air temperature: https://www.esrl.noaa.gov/psd/data/gridded/data.UDel_AirT_Precip.html; GPCP precipitation: https://www.esrl.noaa.gov/psd/data/gridded/data.gpcp.html; HadISST: https://www.metoffice.gov.uk/hadobs/hadisst/; HadSLP2: https://www.metoffice.gov.uk/hadobs/hadslp2/; AMIP experiment data: http://www.esrl.noaa.gov/psd/repository/alias/facts/).

## Code availability

The analysis codes are available from the corresponding author upon reasonable request. The GFDL AM2.1 model code is available as part of the MOM5 project at https://mom-ocean.github.io. All graphics were produced using GrADS version 2.1.0 (http://cola.gmu.edu/grads/).

## References

[1] Cohen J (2014). Recent Arctic amplification and extreme mid-latitude weather. Nat. Geosci..

[2] Panagiotopoulos F, Shahgedanova M, Hannachi A, Stephenson DB (2005). Observed trends and teleconnections of the Siberian high: a recently declining center of action. J. Clim..

[3] Jeong JH (2011). Recent recovery of the Siberian high intensity. J. Geophys. Res..

[4] Zhang X, Lu C, Guan Z (2012). Weakened cyclones, intensified anticyclones and recent extreme cold winter weather events in Eurasia. Environ. Res. Lett..

[5] Overland JE (2015). The melting Arctic and midlatitude weather patterns: are they connected?. J. Clim..

[6] Mori M, Watanabe M, Shiogama H, Inoue J, Kimoto M (2014). Robust Arctic sea-ice influence on the frequent Eurasian cold winters in past decades. Nat. Geosci..

[7] Kug JS (2015). Two distinct influences of Arctic warming on cold winters over North America and East Asia. Nat. Geosci..

[8] Perlwitz J, Hoerling M, Dole R (2015). Arctic tropospheric warming: causes and linkages to lower latitudes. J. Clim..

[9] Sun L, Perlwitz J, Hoerling M (2016). What caused the recent “Warm Arctic, Cold Continents” trend pattern in winter temperatures?. Geophys. Res. Lett..

[10] McCusker KE, Fyfe JC, Sigmond M (2016). Twenty-five winters of unexpected Eurasian cooling unlikely due to Arctic sea-ice loss. Nat. Geosci..

[11] Screen JA, Deser C, Simmonds I (2012). Local and remote controls on observed Arctic warming. Geophys. Res. Lett..

[12] Kosaka Y, Xie S-P (2013). Recent global-warming hiatus tied to equatorial Pacific surface cooling. Nature.

[13] Kosaka Y, Xie S-P (2016). The tropical Pacific as a key pacemaker of the variable rates of global warming. Nat. Geosci..

[14] Ding Q (2014). Tropical forcing of the recent rapid Arctic warming in northeastern Canada and Greenland. Nature.

[15] Enfield DB, Mayer DA (1997). Tropical Atlantic sea surface temperature variability and its relation to El Niño‒Southern Oscillation. J. Geophys. Res..

[16] Klein SA, Soden BJ, Lau N-C (1999). Remote sea surface temperature variations during ENSO: evidence for a tropical atmospheric bridge. J. Clim..

[17] Schott FA, Xie S-P, McCreary JP (2009). Indian Ocean circulation and climate variability. Rev. Geophys..

[18] Xie S-P (2009). Indian Ocean capacitor effect on Indo–western Pacific climate during the summer following El Niño. J. Clim..

[19] Matsuno T (1966). Quasi-geostrophic motions in the equatorial area. J. Meteor. Soc. Jpn.

[20] Gill AE (1980). Some simple solutions for heat-induced tropical circulation. Quart. J. R. Meteor. Soc..

[21] Sorokina SA, Li C, Wettsten JJ, Kvamstø NG (2016). Observed atmospheric coupling between Barents Sea ice and the Warm-Arctic Cold-Siberia anomaly pattern. J. Clim..

[22] Matsumura S, Zhang X, Yamazaki K (2014). Summer Arctic atmospheric circulation response to spring Eurasian snow cover and its possible linkage to accelerated sea ice decrease. J. Clim..

[23] Czaja A, Frankignoul C (2002). Observed impact of Atlantic SST anomalies on the North Atlantic oscillation. J. Clim..

[24] Peng S, Robinson WA, Li S, Hoerling MP (2005). Tropical Atlantic SST forcing of coupled North Atlantic seasonal responses. J. Clim..

[25] Hartmann DL (2015). Pacific sea surface temperature and the winter of 2014. Geophys. Res. Lett..

[26] Di Lorenzo E, Mantua N (2016). Multi-year persistence of the 2014/15 North Pacific marine heatwave. Nat. Clim. Change.

[27] Mori M, Kosaka Y, Watanabe M, Nakamura H, Kimoto M (2019). A reconciled estimate of the influence of Arctic sea-ice loss on recent Eurasian cooling. Nat. Clim. Change.

[28] Smoliak BV, Wallace JM (2015). On the leading patterns of Northern Hemisphere sea level pressure variability. J. Atmos. Sci..

[29] Deser C, Simpson IR, McKinnon KA, Phillips AS (2017). The northern hemisphere extra-tropical atmospheric circulation response to ENSO: how well do we know it and how do we evaluate models accordingly?. J. Clim..

[30] Meehl GA, Chung CTY, Arblaster JM, Holland MM, Bitz CM (2018). Tropical decadal variability and the rate of Arctic sea ice decrease. Geophys. Res. Lett..

[31] Kim BM (2014). Weakening of the stratospheric polar vortex by Arctic sea-ice loss. Nat. Commun..

[32] Giannini A, Kushnir Y, Cane MA (2000). Interannual Variability of Caribbean Rainfall, ENSO, and the Atlantic Ocean. J. Clim..

[33] Sato K, Inoue J, Watanabe M (2014). Influence of the Gulf Stream on the Barents Sea ice retreat and Eurasian coldness during early winter. Environ. Res. Lett..

[34] Petoukhov V, Semenov VA (2010). A link between reduced Barents–Kara sea ice and cold winter extremes over northern continents. J. Geophys. Res..

[35] Okumura YM, Deser C (2010). Asymmetry in the duration of El Niño and La Niña. J. Clim..

[36] Screen JA, Francis JA (2016). Contribution of sea-ice loss to Arctic amplification is regulated by Pacific Ocean decadal variability. Nat. Clim. Change.

[37] Kobayashi S (2015). The JRA-55 Reanalysis: General specifications and basic characteristics. J. Meteor. Soc. Jpn..

[38] Dee DP (2011). The ERA-Interim reanalysis: configuration and performance of the data assimilation system. Q. J. R. Meteorol. Soc..

[39] Saha S (2010). 2010: The NCEP Climate Forecast System Reanalysis. Bull. Am. Meteorol. Soc..

[40] Rayner NA (2003). Global analyses of sea surface temperature, sea ice, and night marine air temperature since the late nineteenth century. J. Geophys. Res..

[41] Huffman GJ, Adler RF, Bolvin DT, Gu G (2009). Improving the global precipitation record: GPCP version 2.1. Geophys. Res. Lett..

[42] Willmott CJ, Matsuura K (1995). Smart interpolation of annually averaged air temperature in the United States. J. Appl. Meteorol..

[43] Allan R, Ansell T (2006). A New Globally Complete Monthly Historical Gridded Mean Sea Level Pressure Dataset (HadSLP2): 1850–2004. J. Clim..

[44] Takaya K, Nakamura H (2001). A formulation of a phase-independent wave-activity flux for stationary and migratory quasigeostrophic eddies on a zonally varying basic flow. J. Atmos. Sci..

