# Supplementary material for: Arctic–Eurasian climate linkage induced by tropical ocean variability
Source: Nat Commun. 2019 Aug 1;10:3441. doi: 10.1038/s41467-019-11359-7 (PMC6672006; doi:10.1038/s41467-019-11359-7)
Supplement: Supplementary file 1 — Supplementary Information [file 41467_2019_11359_MOESM1_ESM.pdf]

Supplementary Information

Arctic–Eurasian climate linkage induced by tropical ocean variability

Matsumura et al.

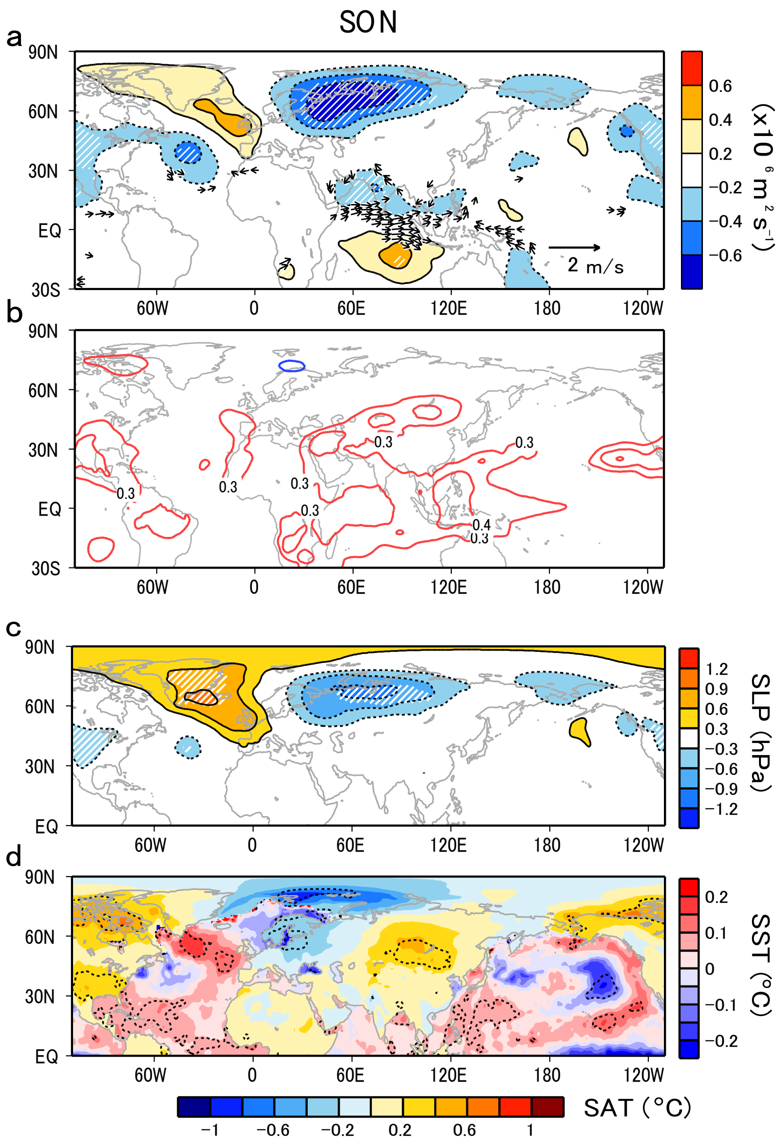

Supplementary Figure 1 Observed climate anomalies in El Niño-decay year. (a) Regressed anomalies of 850-hPa stream function (shading; $\times10^6$ m^2 s^{-1}) and wind velocity (vectors; m s^{-1}) in September–November (SON) onto the ENSO index in preceding winter (ENSO(–1)). (b) As in (a), but for tropospheric temperature (850–250 hPa) correlation. (c), (d) As in (a), but for sea level pressure (SLP) (c), surface air temperature (SAT) and sea surface temperature (SST) (d). Hatching in (a, c) and black dotted contours in (d) indicate statistical significance at the $p < 0.05$ level. Correlation of 0.3 in (b) reaches statistical significance at the $p < 0.05$ level.

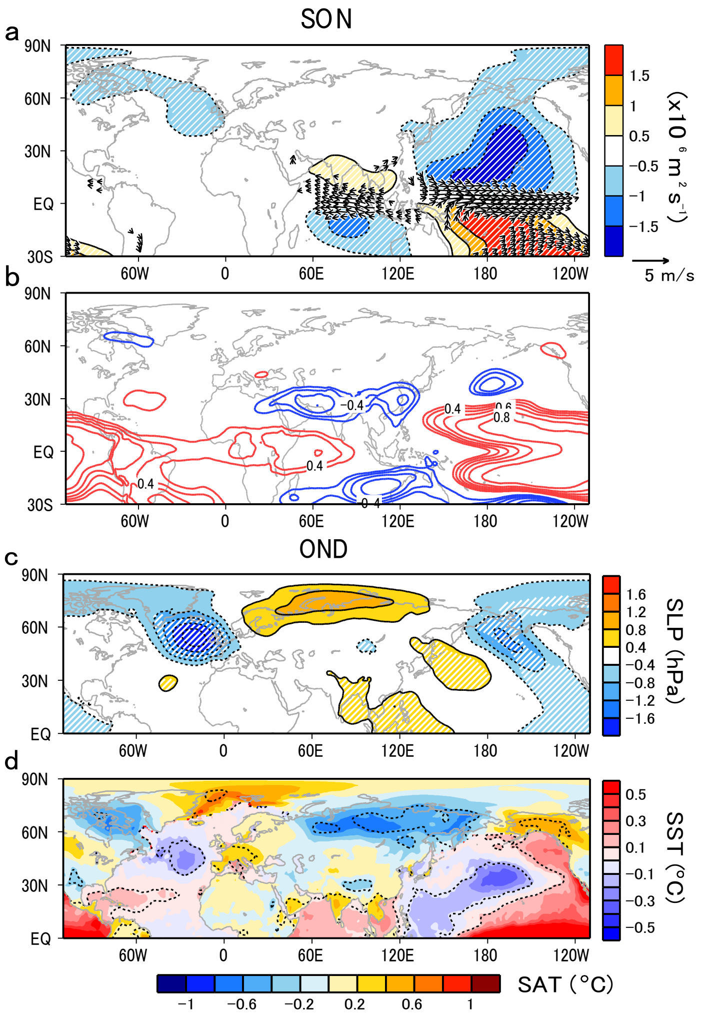

Supplementary Figure 2 Observed climate anomalies in El Niño-developing year. (a) Regressed anomalies of 850-hPa stream function (shading; $\times 10^6$ m^2 s^{-1}) and wind velocity (vectors; m s^{-1}) in September–November (SON) onto ENSO(0). (b) As in (a), but for tropospheric temperature (850–250 hPa) correlation. (c), (d) As in (a), but for sea level pressure (SLP) (c), surface air temperature (SAT) and sea surface temperature (SST) (d) in October–December (OND). Hatching in (a, c) and black dotted contours in (d) indicate statistical significance at the $p < 0.05$ level. Correlation of 0.3 in (b) reaches statistical significance at the $p < 0.05$ level.

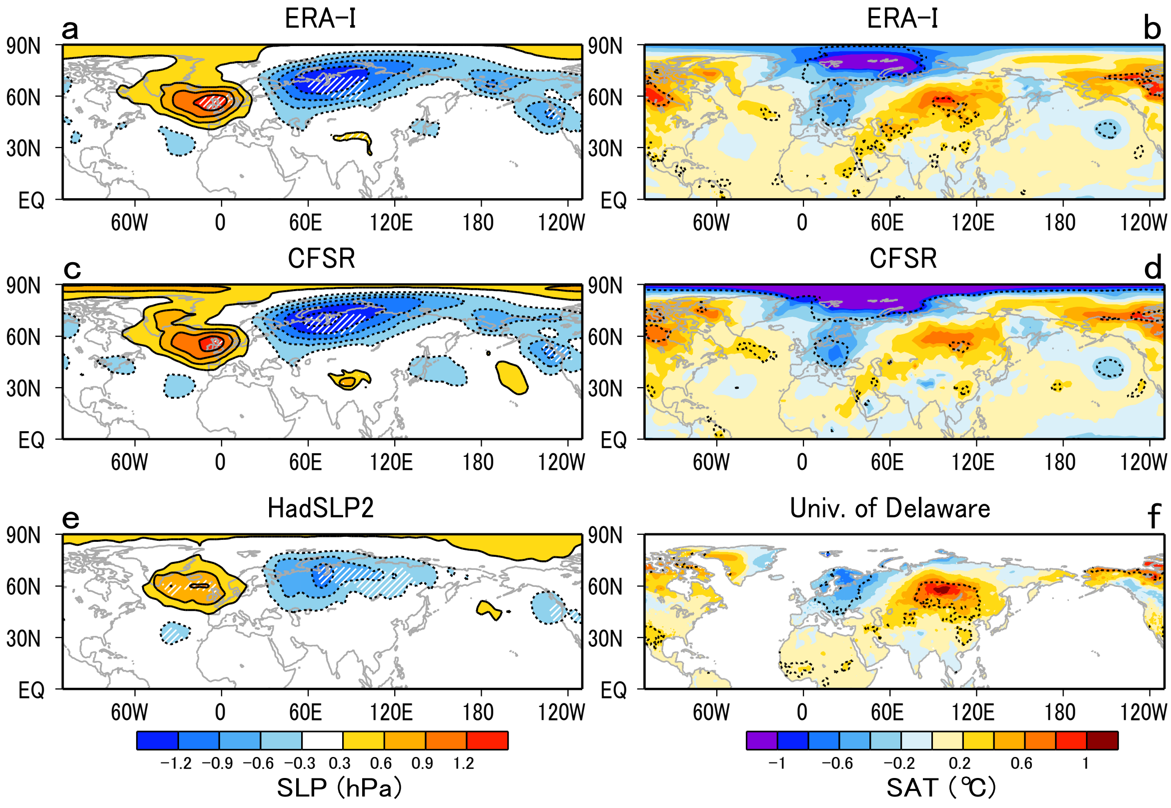

Supplementary Figure 3 Robustness of observed climate anomalies in El Niño-decay year. Regressed anomalies of (a) sea level pressure (SLP) and (b) surface air temperature (SAT) in early winter onto ENSO(–1) for 1979–2015, based on ERA-Interim. (c), (d) As in (a) and (b), but for CFSR for 1979–2010. (e) As in (a), but for HadSLP2 for 1958–2014. (f) As in (b), but for University of Delaware for 1958–2014.

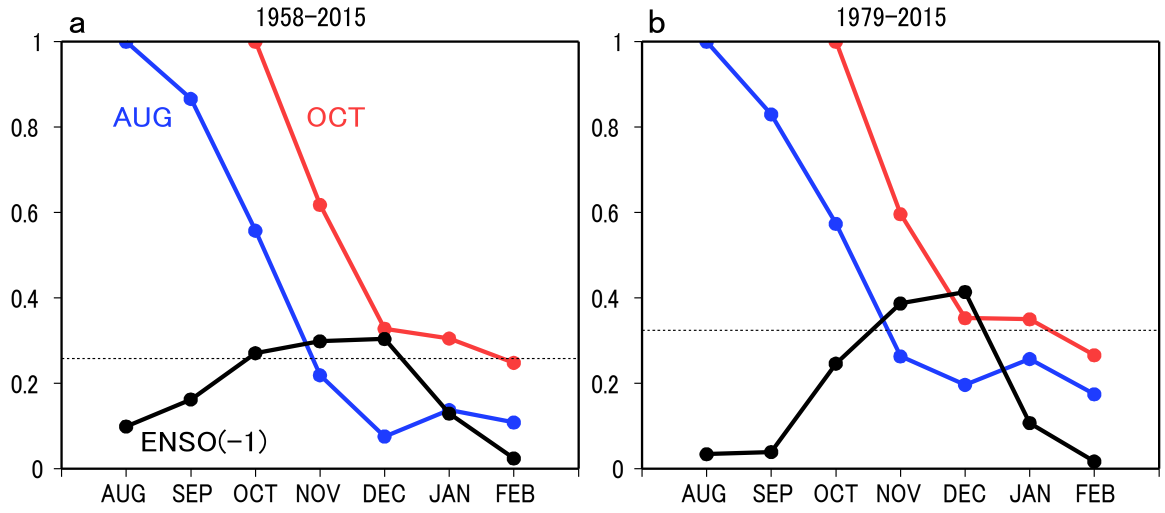

Supplementary Figure 4 Seasonal evolution of sea ice concentration in Barents–Kara Seas. Autocorrelation of sea ice concentration averaged in the Barents–Kara Sea in August (blue) and October (red), and its correlation with ENSO(–1) (black) from August to February for the period (a) 1958–2015 and (b) 1979–2015. Dotted lines indicate statistical significance at the $p < 0.05$ level.

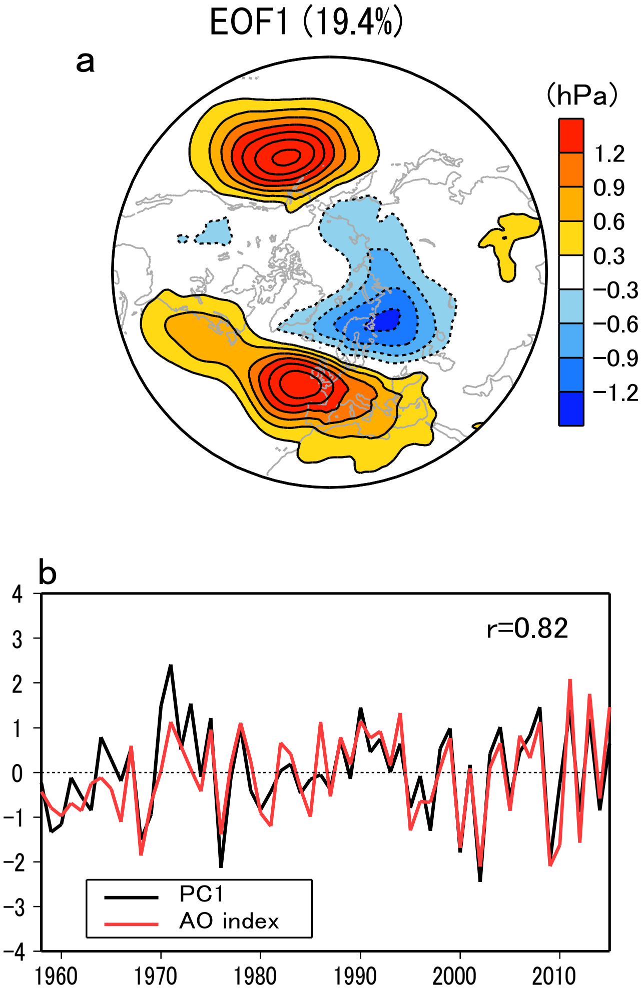

Supplementary Figure 5 The leading mode of Northern Hemisphere circulation in early winter. (a) First EOF mode of sea level pressure in early winter and (b) corresponding principal component (PC) for 1958–2015. Red line in (b) shows the Arctic oscillation (AO) index obtained from the NOAA Climate Prediction Center.

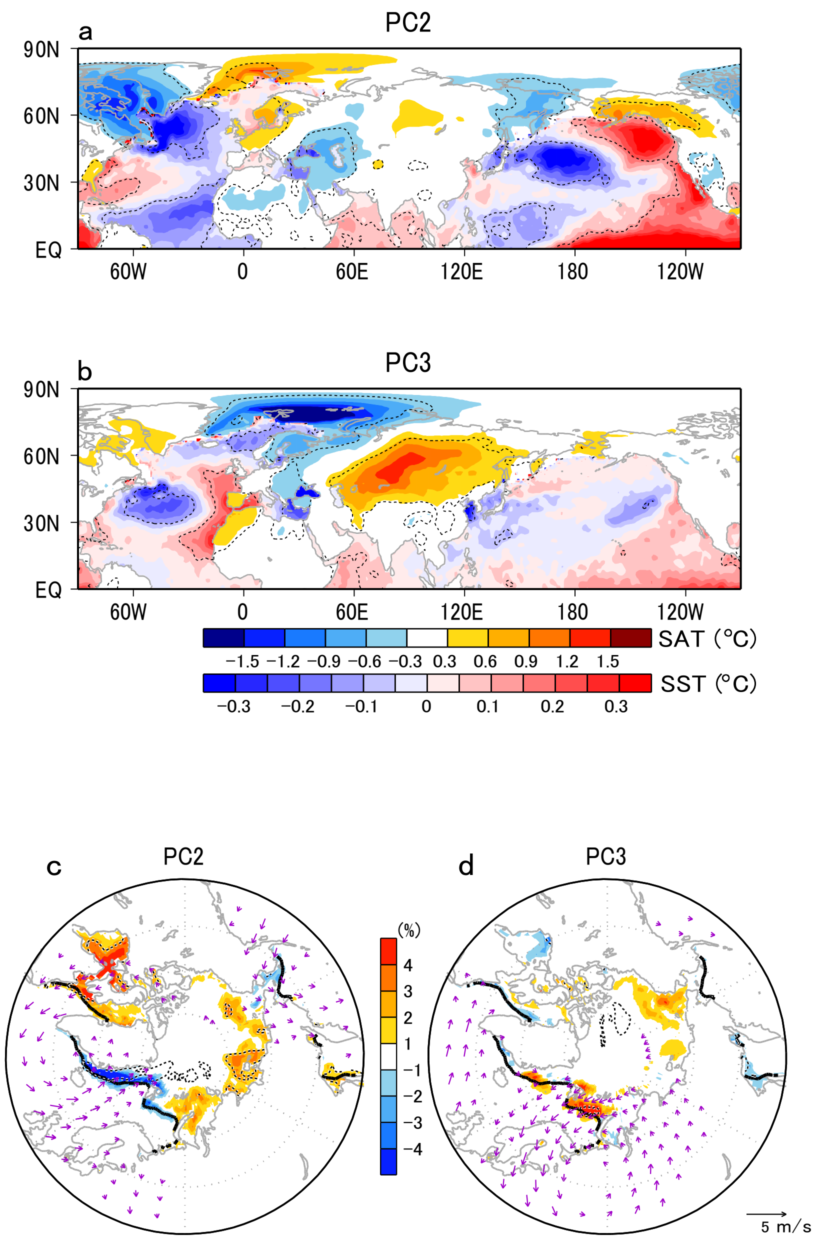

Supplementary Figure 6 Surface climate anomalies associated with two major atmospheric modes. Regressed anomalies of surface air temperature (SAT) and sea surface temperature (SST) onto principal components (PCs) of the (a) second and (b) third EOF modes. (c), (d) As in (a) and (b), but for sea ice concentration (SIC) and 925-hPa wind velocity (vectors; limited to 50°–82°N). Dotted contours indicate statistical significance at the $p < 0.05$ level and thick black contours in (c, d) indicate mean SIC of 10%.

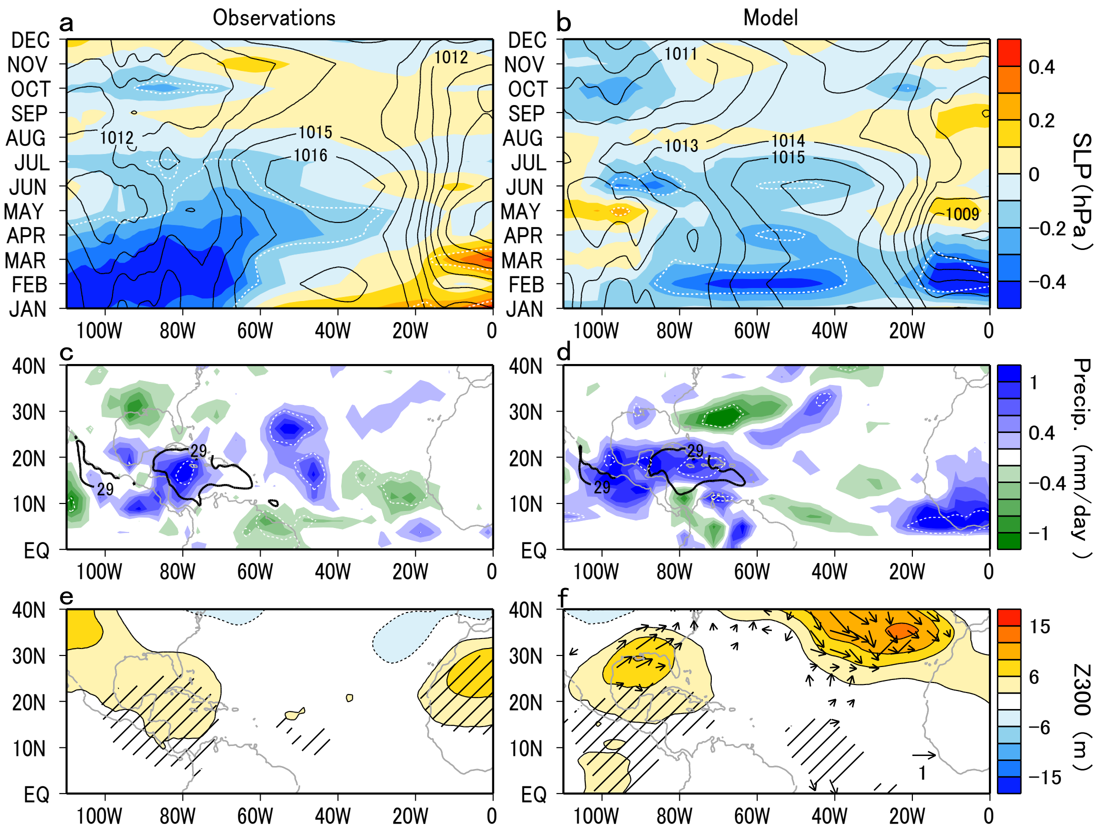

Supplementary Figure 7 Atmospheric response to tropical Atlantic warming in October. (a) Longitude–time section of sea level pressure (SLP) anomalies over 10°–20°N regressed onto ENSO(–1). The time evolution is from January to December of ENSO-decaying year. Regressed anomalies of (c) GPCP precipitation (1982–2015) and (e) 300-hPa geopotential height (Z300) in October onto ENSO(–1). (b), (d), and (f) As in (a), (c), and (e), but for ATL + ICE (tropical North Atlantic warming and Arctic sea ice increase) – CTL (control experiment) difference. Black contours indicate (a) mean SLP (contour interval is 1 hPa), and October mean sea surface temperature of 29°C for (c) 1982–2015 and for (d) 1958–2015. Vectors in (f) indicate wave activity fluxes (m^2 s^{-2}). White dotted contours and hatching indicate statistical significance at the $p < 0.05$ level.

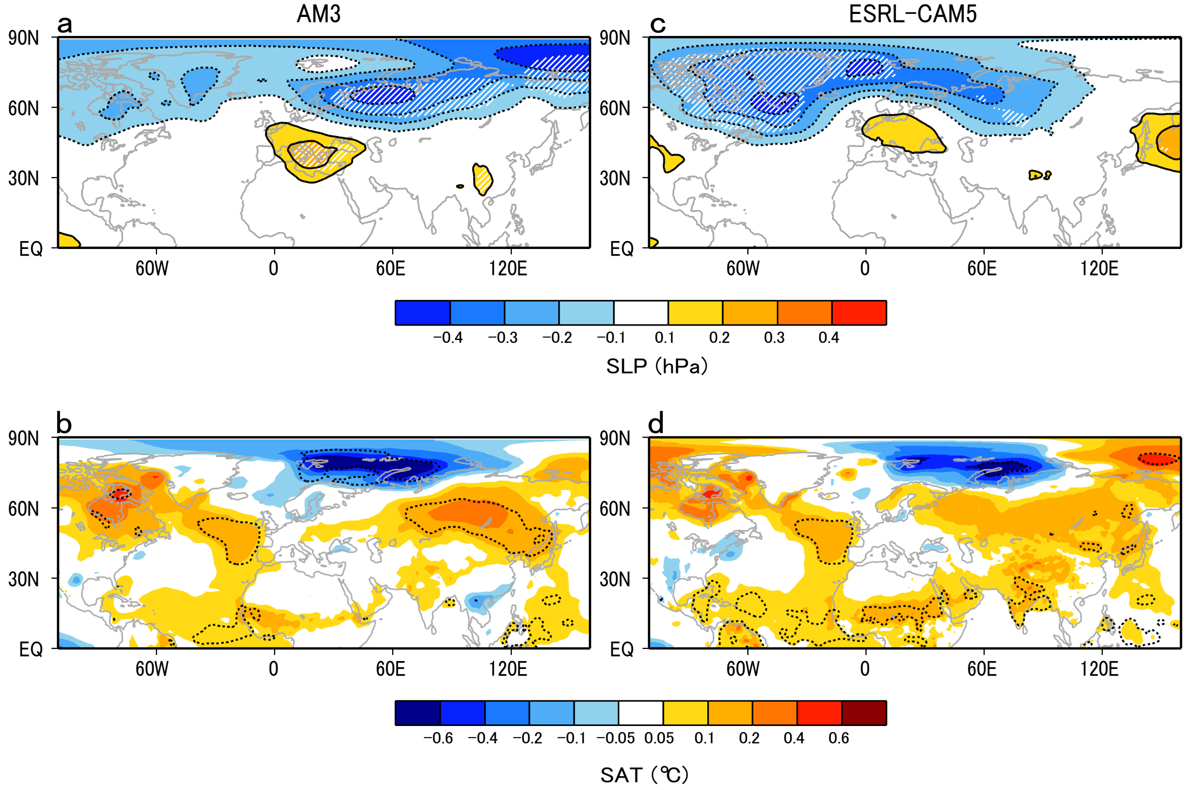

Supplementary Figure 8 Additional modelling evidence. Regressed anomalies of sea level pressure (SLP) onto ENSO(–1) in (a) GFDL AM3 (1958–2014) and (c) ESRL-CAM5 (1958–2015). (b), (d) As in (a) and (c), but for surface air temperature (SAT). Hatching and dotted contours in (b, d) indicate statistical significance at the $p < 0.05$ level.

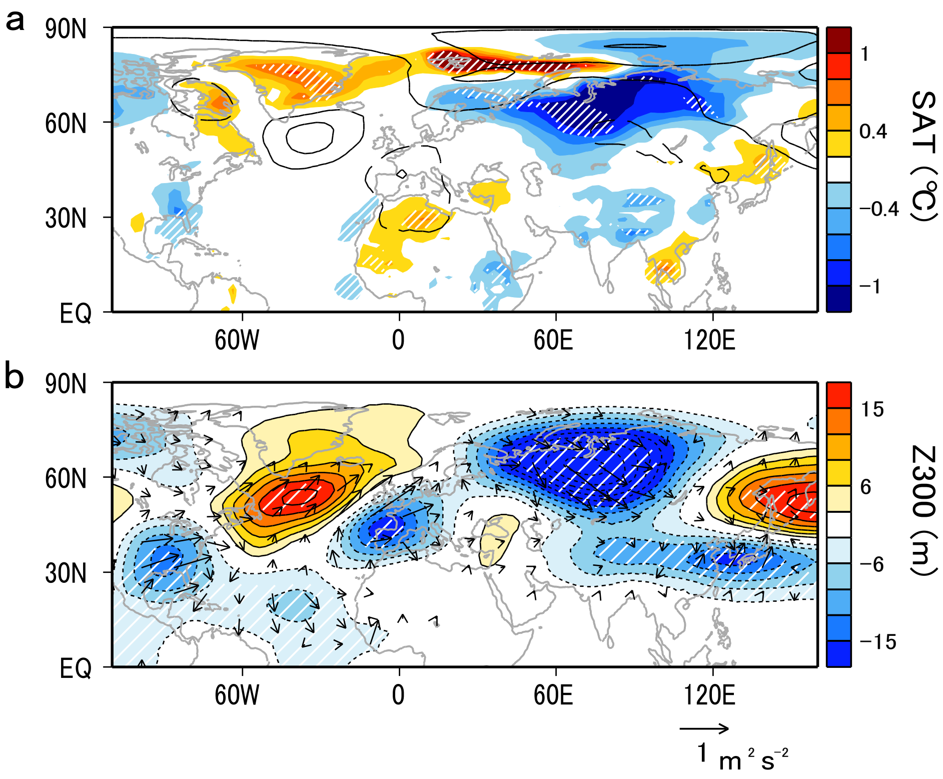

Supplementary Figure 9 Simulated atmospheric responses to tropical Atlantic cooling and Arctic sea ice decrease. (a) Surface air temperature (SAT) and sea level pressure (SLP) (for ±0.5, ±1, … hPa; solid for positive and dashed for negative) difference between the negative and positive ATL + ICE experiments (negative: tropical North Atlantic cooling and Arctic sea ice decrease, positive: tropical North Atlantic warming and Arctic sea ice increase). (b) As in (a), but for 300-hPa geopotential height (Z300) and wave activity fluxes (vectors; m^2 s^{-2}). Hatching indicates statistical significance at the $p < 0.05$ level.

Supplementary Table 1. Correlation coefficients between ENSO index and principal components (PCs).

	ENSO(0)	ENSO(–1)
**PC2**	0.44**	–0.13
**PC3**	0.15	0.23*

* Correlation is statistically significant at the 90% confidence level, and ** Correlation is at the 99% level.
